# Thomas John Wydrzynski (8 July 1947–16 March 2018)

**DOI:** 10.1007/s11120-018-0606-9

**Published:** 2018-11-26

**Authors:** Brendon Conlan, Govindjee, Johannes Messinger

**Affiliations:** 1 Research School of Biological Sciences, Australian Capital Territory, Australian National University, Acton, ACT 0200 Australia; 2 Department of Plant Biology, Department of Biochemistry, Center of Biophysics & Quantitative Biology, University of Illinois at Urbana-Champaign, Urbana, IL 61801 USA; 3 Department of Chemistry – Ångström, Uppsala University, Lägerhyddsvägen 1, 75120 Uppsala, Sweden; 4 Department of Chemistry, Umeå University, Linnaeus väg 6, 90187 Umeå, Sweden

**Keywords:** Water oxidation, Photosystem II, Manganese, Chloride, Artificial photosynthesis

## Abstract

With this Tribute, we remember and honor Thomas John (Tom) Wydrzynski. Tom was a highly innovative, independent and committed researcher, who had, early in his career, defined his life-long research goal. He was committed to understand how Photosystem II produces molecular oxygen from water, using the energy of sunlight, and to apply this knowledge towards making artificial systems. In this tribute, we summarize his research journey, which involved working on ‘soft money’ in several laboratories around the world for many years, as well as his research achievements. We also reflect upon his approach to life, science and student supervision, as we perceive it. Tom was not only a thoughtful scientist that inspired many to enter this field of research, but also a wonderful supervisor and friend, who is deeply missed (see footnote*).

## Tom’s life

**Fig. 1:**
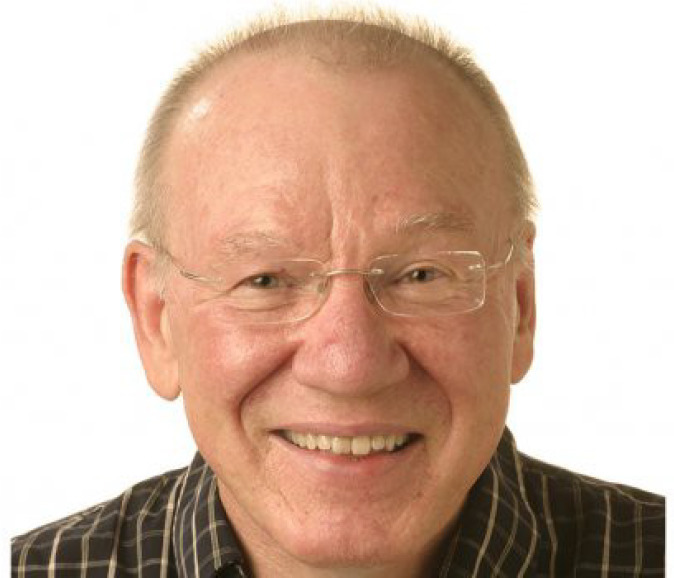
Tom Wydrzynski. Credit Research School of Biology, ANU, Canberra. 2009

**Fig. 2:**
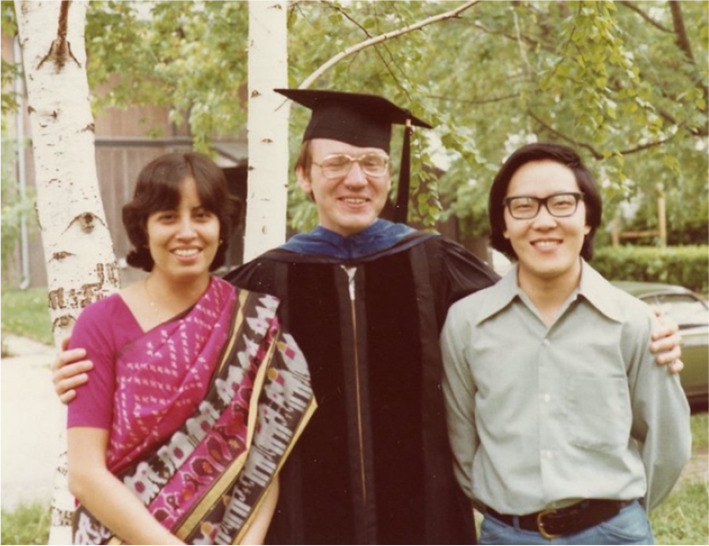
Having fun: Tom in the graduation robe of Rita Khanna (left) at her PhD graduation in May 1980 at the University of Illinois at Urbana-Champaign. Both Tom and Daniel Wong (to the right) had graduated earlier

Figure 1 shows a portrait of Tom Wydrzynski. Tom was born in St Louis, Missouri, USA, as the first of four children of Eva and Stanley Wydrzynski. Tom graduated in 1969 with a degree in biology and chemistry from the University of Missouri, St Louis. He then studied plant physiology at Queens University, Kingston, Ontario, Canada, before entering graduate school at the University of Illinois, Urbana-Champaign (UIUC) in 1972, with glowing recommendations by his former teachers. They described him as follows: ‘‘Tom has certainly the guts to go after what he wants….’’; “intelligent”; “conscientious”; and “one of the most industrious and conscientious students I have come across.’’ Tom completed his PhD under the tutelage of one of us (Govindjee) in 1977. Figure 2 shows Tom after the graduation of Rita Khanna, in 1980, in her graduation robe together with their lab mate Daniel Wong. Tom’s initiation and early work in photosynthesis has been covered in detail by Govindjee (2008) and in Govindjee’s 2018 “Eulogy” (Govindjee et al. (2018).

**Fig. 3:**
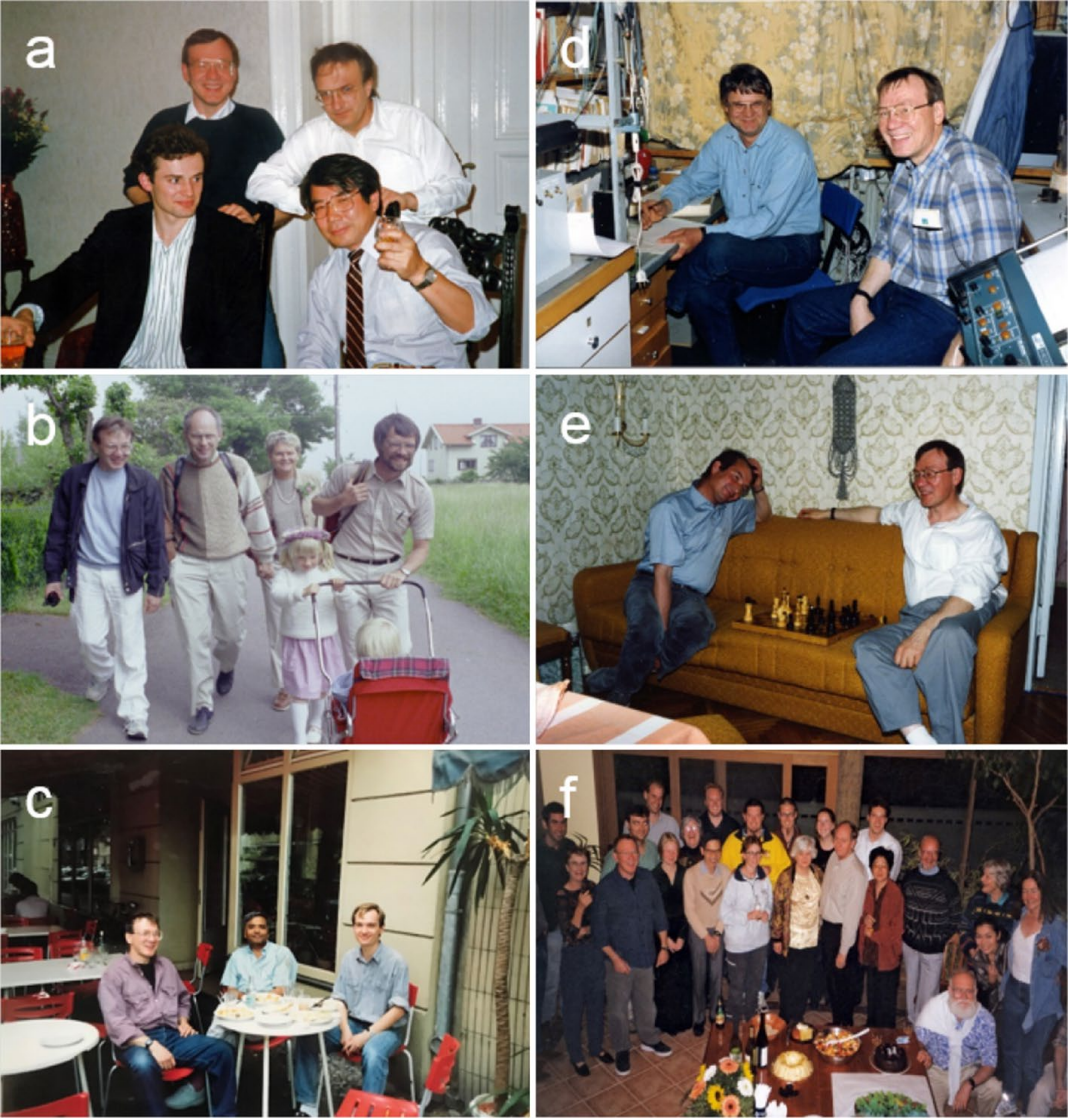
Photographs of Tom with others. **a** Standing (left to right): Tom and Gernot Renger; sitting (left to right): Hermann Gleiter and Yorinao Inoue (date unknown, Japan) **b** Midsummer celebrations on the island of Grötö, outside Gothenburg (1985, Sweden): Tom with Lars-Erik Andreasson (to his extreme right with his daughters) and in the center are Catharina and Karl-Erik Falk. **c** Tom, Govindjee and Johannes at an Indian restaurant in Berlin, 1989. **d** Gennady Ananyev and Tom in the lab of Slava Klimov in Pushchino (Russia) in 1990. **e** Slava Klimov and Tom playing chess in Pushchino (1990). **f** Tom at a celebration at his home in Canberra with Brett Wallace (back 1st from left) Karin Åhrling (front, 1st from left), Fred Chow (front, 4th from left), Jan Anderson (2nd row to the left of Fred Chow) and Barry Osmond (front, 7th from left) and many others

After finishing his PhD degree, Tom held two post-doctoral positions in leading photosynthesis labs. First he spent 4 months in the lab of Jean-Marie Briantais and Ann-Lise Etienne in Gif-sur-Yvette & Paris, France, and then he joined the lab of Kenneth (Ken) Sauer for 2 years (1977–1979) at the University of California, Berkeley. Subsequently, Tom worked for 4 years (1980–1984) as a Research Chemist at the Standard Oil Company. Here he led a small team that had the goal to increase the knowledge of the light reactions in photosynthesis and, from this basis, to develop artificial photosynthetic systems. While Standard Oil was initially highly supportive of this visionary basic research, in 1984, it became clear that he should move to other research areas within the company. For Tom there was only one choice: to quit his well-paid company position in order to continue his research track on *water oxidation in photosystem II*. This started a period of nearly 7 years (1985–1991) on ‘soft’ research money. Thanks to receiving a prestigious one-year Humboldt fellowship, he was able to associate with the research group of Gernot Renger (Siggel et al. 2016) at the Technical University (TU) Berlin in Germany. This first period in Berlin was split into two blocks by a 4-month research visit to Yorinao Inoue at Rikagaku Kenkyūjyo (RIKEN), Japan, which was financed by a Japanese Versailles fellowship. Figure 3a shows Tom with Gernot Renger and Yorinao Inoue. After finishing the second part of his Humboldt fellowship in Berlin, he joined Tore Vänngård at Chalmers University in Gothenburg, in 1986, on the basis of a 2-year Wennergren fellowship, where he also met Lars-Erik Andreasson (Fig. 3b). In 1988, he returned to Gernot Renger’s group in Berlin where he was financed by a DFG (Deutsche Forschungsgemeinschaft) project. It was during Tom’s final period in Berlin, that Johannes (author of this paper) first met Tom while doing his PhD in Gernot Renger’s lab. Frequently, they and others from the lab had lunch at various Indian restaurants in the area around the Technische Universität Berlin. Figure 3c shows Tom and Johannes with Govindjee, during one of Gov’s visits to Berlin. This time also included research visits to the lab of Slava Klimov in Pushchino, Russia (Allakhverdiev et al. 2018). Figure 3d, e show Tom with Gennady Ananyev in the lab in Pushchino and playing chess with Slava Klimov in his home.

It was only in 1991, and after having worked in nine different short term positions or fellowships, that Barry Osmond, at the time director of the Research School of Biological Sciences (RSBS) at the Australian National University (ANU) in Canberra, Australia, employed Tom as the head of an initially small research team working on the biophysical aspects of photosynthesis. Barry Osmond’s aim was to continue the strong position of Australia in this area that was established, among others, by Keith Boardman and Jan Anderson (Chow et al. 2016). In Australia, where Tom made his new home, he started as a non-tenured Fellow, but subsequently climbed the ranks to become Professor in 2007. Figure 3f shows Tom at a celebration at his home in Canberra with Barry Osmond, Jan Anderson and many others. In 2011, after 20 years in Australia, Tom retired from ANU, and moved back to St. Louis, USA, while remaining a Visiting Fellow at ANU until August 2017. In St Louis, he received loving support from his brothers and sister and their families, and together they visited the International Congress on Photosynthesis held in St. Louis in 2013, where most of us saw Tom for the last time. Due to health problems, which in part originated from a childhood polio infection, he remained largely private until he died, at age 71, on March 16, 2018.

## Tom’s research path

**Fig. 4:**
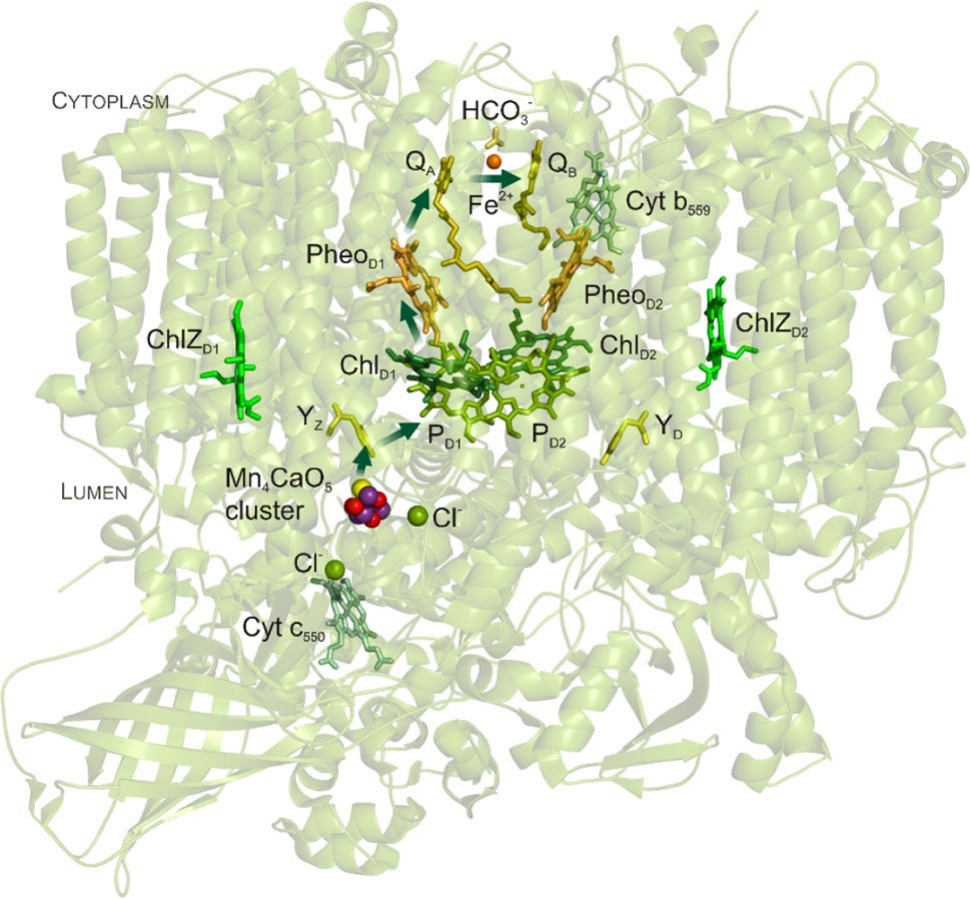
Structure of Photosystem II and some important redox cofactors. Modified from Shevela and Messinger (2013)

Tom’s first PhD project (1974–1975), in collaboration with Prasanna Mohanty, was to investigate the role of magnesium and sodium in regulating photosynthesis. While Tom completed and published this work, it clearly was not his passion (Mohanty et al. 1974; Wydrzynski et al. 1975a). Upon the suggestion of Govindjee, Tom turned to solving the problem of where precisely bicarbonate/CO_2_ functions in the steps of electron transport from water to a specific electron carrier. While the assertion by the 1931 Nobel laureate Otto Warburg that bicarbonate was the source of oxygen in photosynthesis was by that time no longer accepted (see e.g., Clausen et al. 2005; Hillier et al. 2006; McConnell et al. 2007), there were indications that bicarbonate nevertheless did have an obligatory role in the electron transfer part of photosynthesis. Tom’s breakthrough discovery, in 1975, was that bicarbonate has a function in electron transfer on the acceptor side of photosystem II (Wydrzynski and Govindjee 1975). This work inspired many subsequent studies by several other students of Govindjee and by scholars around the world that hotly debated donor vs acceptor side effects of bicarbonate (Khanna et al. 1977). Figure 4 shows a schematic of the photosystem II complex indicating the binding of bicarbonate at the acceptor side of PSII, which in the meantime has been confirmed by X-ray crystallography (Umena et al. 2011). Recent results demonstrate that bicarbonate has a function on both sites of photosystem II (Shevela et al. 2012; Koroidov et al. 2014; Brinkert et al. 2016).

In spite of his success with research in Govindjee’s lab, Tom desired his ‘own’ project and announced to Govindjee that he wanted to work on “How Plants Make Oxygen”, and that he would attack the problem by using Nuclear Magnetic Resonance (NMR), something no one had done before in photosynthesis. For this, he collaborated with Paul G. Schmidt and Herb Gutowsky on NMR, ESR and EXAFS experiments on Photosystem II. Tom published three important papers on what was learned from this innovative application of NMR to probe water oxidation (Wydrzynski et al. 1975b, 1976, 1978). He was the first to show that period 4 oscillations occur in NMR relaxation, which is consistent with the period 4 oscillations in oxygen evolution, as discovered only a few years earlier by Pierre Joliot (Joliot et al. 1969). Tom later summarized his and others early work on the involvement of Mn in water oxidation in an excellent mini review (Wydrzynski 2004).

Being interested in water oxidation in PSII, a research stay in Paris, the place where Pierre Joliot had discovered the period-four O_2_ oscillation pattern, was a logical next step after completing his PhD. In the 4-month stay in the labs of Jean-Marie Briantais and Ann-Lise Etienne, he continued his early work on cation effects on photosystem II (Govindjee et al. 1979) and also worked on using a short heat treatment to release Mn from broken thylakoids. Tom then worked with Kenneth Sauer at the Melvin Calvin Laboratory, University of California, Berkeley, where he used this experience on heat-induced Mn release in an attempt to determine the stoichiometry and oxidation states of the manganese ions, which he detected by Electron Paramagnetic Resonance (EPR) and other techniques (Wydrzynski and Sauer 1980; Kirby et al. 1981). Tom concluded that Mn was oxidized in the S_0_→S_1_ and S_1_→S_2_ transitions, but not in the S_2_→S_3_ transition. The latter idea, which was consistent with his earlier NMR data, set the scene for a long-standing discussion in the field that now appears to be resolved; nearly all groups accept Mn^III^ to Mn^IV^ oxidations occurring in all S state transitions up the S_3_ state (Haumann et al. 2005; Cox et al. 2014; Krewald et al. 2015). In 1982, Tom wrote an invited thorough chapter on the process of water oxidation as was known at that time (Wydrzynski 1982). Over the years Tom published a number of further insightful reviews on water oxidation in photosystem II and edited two books (Hansson and Wydrzynski 1990; Wydrzynski and Renger 1990; Renger and Wydrzynski 1991; Wydrzynski et al. 1996; Hillier and Wydrzynski 2001; Wydrzynski and Satoh 2005; Wydrzynski 2008; Wydrzynski and Hillier 2011).

In his 4 years of working in industry (see above), he had the ambition to further the understanding of *light reactions in photosynthesis* in order to apply it to develop artificial photosynthetic systems. His small team had “perturbed” PSII with a large number of different chemicals, including various solvents, detergents and a variety of other agents. In one such study, he reported the effects of methyl ethyl ketone (MEK) and lauryl choline chloride (LCC) (Wydrzynski and Huggins 1983). LCC was found to decouple oxygen evolution from electron flow through PSII (Wydrzynski et al. 1985). In Gernot Renger’s lab, Tom continued to study the effect of LCC, but its interaction with PSII still remains to be fully understood (Eckert et al. 1988). In continuation of his early NMR proton relaxation work, Tom also studied in Berlin the effects of the chelating agent EDTA (**e**thylene **d**iamine **t**etra **a**cetic acid) on proton relaxation (Wydrzynski and Renger 1986). This work indicated a direct interaction of EDTA with the Mn-cluster, in addition to the binding of free Mn^2+^. Furthermore, Tom started to investigate the role of chloride (Cl^−^) in PSII using Cl^−^ NMR (Wydrzynski et al. 1990a). In the lab of Yorinao Inoue, Tom returned briefly to his earlier work on the acceptor side of photosystem II and studied the binding of various quinones to PSII using thermoluminescence (Wydrzynski and Inoue 1987).

In Gothenburg, Sweden, Tom worked with Tore Vänngård and collaborated later with Lars-Erik Andreasson, whom he met during this period (Fig. 3b). In Gothenburg he discovered that certain perturbations of PSII can lead to light-induced peroxide formation at the expense of O_2_ evolution (Wydrzynski et al. 1989). In addition, he continued the work on the Cl^−^ effect using Cl^−^ NMR and radioactive Cl^−^, demonstrating that Cl^−^ release coincides with Mn release from the PSII reaction center and that only a single Cl^−^ binding site exists (Lindberg et al. 1990; Wydrzynski et al. 1990b). Together with Örjan Hansson, who had just returned to Gothenburg from a post-doc with Paul Mathis (in France), Tom wrote a highly influential review on water oxidation in PSII (Hansson and Wydrzynski 1990).

Back in Berlin, Tom continued the work on peroxide formation by PSII, and for this, he made two trips to Pushchino, Russia, to work with Gennady Ananyev in Slava Klimov’s lab who had developed a special membrane covered Clark-type electrode sensitive enough for measuring flash-induced O_2_ evolution (Fig. 3d, e). One of us (Johannes), who accompanied Tom in one of his trips to Pushchino, remembers that one of the problems using the membrane-less Joliot-type electrode was to distinguish H_2_O_2_ evolution from O_2_ evolution and to avoid the interaction of H_2_O_2_ produced by the electrode with PSII (Pham and Messinger 2014). Using Gennady’s electrode and chemiluminescence, Tom could show that LCC induces a transient H_2_O_2_ formation (Ananyev et al. 1992; Klimov et al. 1993). On the basis of these experiments, Tom developed the idea that the water accessibility to the Mn-cluster must be regulated by water channels and gates to avoid premature peroxide formation (Wydrzynski et al. 1996). His idea was that the Mn-cluster must stay ‘dry’ until the S_4_ state, where then a conformational change in the protein would allow water binding and oxidation. This was quite different from Gernot Renger’s idea that water is bound to the Mn-cluster in all the S states (Renger 1987, 1997). This scientific controversy was an important stimulus for developing, later (see below), the substrate water exchange experiments using membrane inlet mass spectrometry (Messinger et al. 1995).

**Fig. 5:**
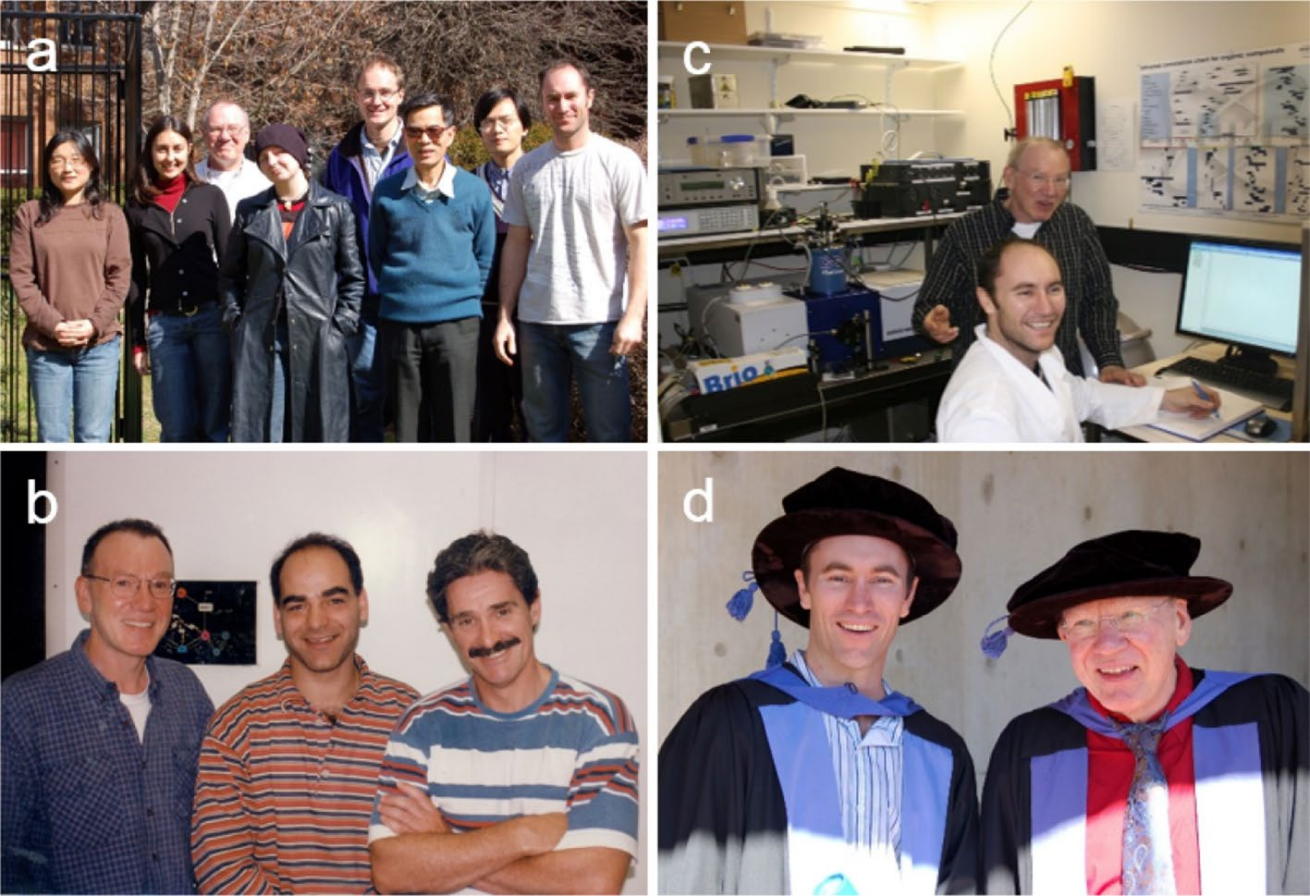
Photographs of Tom with others in Australia. **a** Tom’s research group in August 2007. From left to right Eun-Ha Kim, Sonita Singh, Tom, Adele Williamson, Warwick Hillier, Fred Chow, Dayong Fan, Brendon Conlan. **b** Left to right: Tom, Reza Razeghifard and Ron Pace in Tom’s lab, ca. 1995. **c** Brendon Conlan (one of the authors) with Tom in the lab, 2008 **d** Brendon and Tom at Brendon’s graduation in 2009

As mentioned above, Tom was recruited, in 1991, to the Director’s Research Unit at the Research School of Biological Sciences at ANU. With his first Master student, the late Warwick Hillier (Fig. 5a) (Messinger et al. 2014) Tom investigated the peroxide release of Photosystem II centers deprived of their extrinsic proteins (Hillier and Wydrzynski 1993). In collaboration with G. Fischer, Tom’s first PhD student, Haoming Zhang, employed then FTIR spectroscopy to study the effects of H_2_O_2_ and high light on the protein conformation of PSII (Zhang et al. 1995; Yamamoto et al. 1998). They also obtained time-resolved Fourier Transform Infra Red (FTIR) spectra of the Q_A_ and the Y_Z_ interactions with PSII (Zhang et al. 1997, 1998; Fischer and Wydrzynski 2001).

In 1993, Tom was joined by one of us (Johannes), who arrived right after finishing his PhD to become Tom’s first post-doctoral fellow at the Research School of Biological Science, Australian National University. With support of Murray Badger, Johannes devised a mass spectrometer inlet system that improved the kinetic resolution of substrate water isotope exchange measurements on PSII a thousand-fold compared to previous attempts (Hoch and Kok 1963; Bader et al. 1993; Messinger et al. 1995; Beckmann et al. 2009). With this new technique, the group demonstrated, for the first time, that at least one-substrate water molecule was bound in the S_3_ state of PSII – seemingly a compromise between Tom’s and Gernot Renger’s views. However, a three-fold further improvement of the mixing time, performed by Warwick Hillier, then allowed one to show that indeed both substrate water molecules are bound, in chemically different environments, to the catalytic site of water oxidation in the S_2_ and S_3_ states, and that at least one-substrate water is bound in the S_0_ and S_1_ states (Hillier et al. 1998a, b).

Subsequently, Warwick Hillier, Garth Hendry and others also studied the effects of the removal of the extrinsic proteins, Ca^2+^/Sr^2+^ substitution and of mutants on the substrate water exchange (Hillier and Wydrzynski 2000, 2001, 2004, 2008; Singh et al. 2008; Service et al. 2011; Cox and Messinger 2013). A highly significant finding was the demonstration that Ca^2+^ is involved in the binding and/or exchange of the slowly exchanging substrate water molecule (Hendry and Wydrzynski 2002, 2003). Surprisingly, the removal of the extrinsic proteins led to a slight slowdown of water exchange, rather than the expected increase, demonstrating that any water access restriction would need to be in the vicinity of the Mn-cluster (Hillier et al. 2001).

When moving to RSBS in Canberra (Australia), Tom also had the innovative idea that an intact Mn-cluster or a small protein unit, carrying this cluster, can perhaps be isolated in non-aqueous solvents. Tom’s group thus studied for a while the effects of ethylene glycol on PSII (Hillier et al. 1997).

As head of the group renamed **Photobioenergetics** and strengthened by the move of Jan Anderson and Fred Chow (Figs. 3f, 5b) from CSIRO (Commonwealth Scientific and Industrial Research Organisation) to RSBS, Tom forged strong links with the Department of Chemistry (Ron Pace; see Fig. 5b) and in the Research School of Chemistry (Elmars Krausz). Together they studied the effect of infrared light on the Mn-cluster (Baxter et al. 1999; Fischer and Wydrzynski 2001), employed magneto-optical spectroscopy (Smith et al. 2002; Hughes et al. 2006), and studied the kinetics of Y_Z_, the tyrosine, which is the electron donor to P680 (see Fig. 4), the reaction center of PSII (Razeghifard et al. 1997). During his time at ANU, Tom also took on the task of editing the book “Photosystem II – The Light-driven Water: Plastoquinone Oxidoreductase” with Kimiyuki Satoh (2005), which turned out to be one of the best collections of articles in the field. Continuing with his interest in the manganese center, Adele Williamson within Tom’s lab, completed a number of studies looking at the structure and function of the manganese stabilizing protein (Williamson et al. 2007, 2008; Williamson 2008).

During the last, but highly exciting phase of his research life, Tom turned his attention to one of his early goals: *to engineering of synthetic proteins that, similar to the photosynthetic apparatus, capture light in pigments and employ the excitation energy for charge separation and oxidation*/*reduction of redox-active cofactors*. This work was kicked off with Reza Razeghifard (Fig. 5b) who was able to bind a Zn-chlorin porphyrin molecule within an engineered maquette peptide and show light-induced electron transfer from the chlorin to an acceptor in solution (Razeghifard and Wydrzynski 2003; Razeghifard et al. 2007). Next, Sam Hay, who was a PhD student in Tom’s lab, showed electron transfer from a photoactive Zn-chlorin to a covalently bound quinone within an engineered cytochrome protein (Hay et al. 2004; Hay and Wydrzynski 2005) and again this put Tom’s lab in the spotlight of an exciting new research field. After building this protein containing a light activated pigment and an electron acceptor site, one of us (Brendon Conlan; Fig. 5c, d) attempted to engineer a protein, which contained an electron donor in the form of metal center capable of donating electrons to a photo-oxidized pigment (Wydrzynski et al. 2007; Conlan 2008). Brendon engineered a protein, which bound the photoactive Zinc chlorin and upon illumination was capable of photo-catalytic oxidation of a di-manganese center (Conlan et al. 2009). Tom’s research group then went on to build a light activated protein containing both the metal electron donor site and a quinone acceptor site (Williamson et al. 2011). In a parallel effort, K. Hingorani and Tom engineered bacterioferritin into a ‘reaction center’ (Hingorani et al. 2009, 2014). Together with Warwick Hillier, Tom edited another book, Molecular Solar Fuels (Wydrzynski and Hillier 2011); in this book, Brendon co-authored a chapter with both the editors titled ‘Synthetic Photo-catalytic Proteins—a model of Photosystem II’, which gave an update on the current state of engineering photo-catalytic proteins (Conlan et al. 2012).

## Tom’s personality and approach to supervision

While Tom’s complicated, but unique, career path required endurance and sacrifices, it provided him with an unparalleled research network and experience, both of which benefitted his PhD students and post-docs immensely.

Tom was a good friend and mentor to all his students and post-docs throughout the years and provided many opportunities for them to progress in their careers. Tom was a patient, kind and generous supervisor who was always ready to provide guidance and suggest new approaches when experiments failed. He provided an immensely creative research environment that trained and cultured thinking out-of-the-box, and to follow research tracks outside the mainstream photosynthesis research. He gave his students and post-docs a lot of freedom in choosing and developing their own research projects. Given the ever tighter regulations about PhD supervision in many countries, and the need to produce predefined results in third party funded research projects written by the supervisor, Tom is a role model that we should follow in our supervision as much as possible despite the constraints one may face. Tom Wydrzynski viewed it as one of his greatest achievements that both Johannes Messinger (in 1998 in Budapest) and the late Warwick Hillier (in 2007 in Glasgow) received the triennial Robin Hill award given out by the International Society of Photosynthesis Research.

**Fig. 6:**
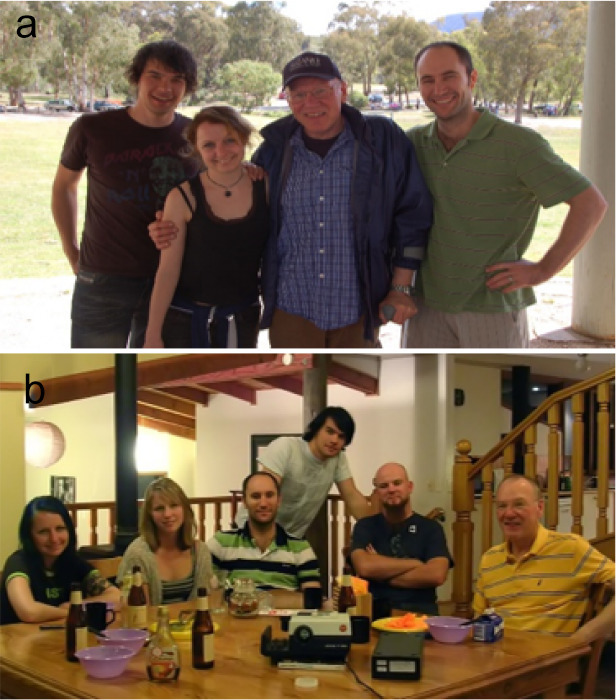
**a** Iain McConnell, Adele Williamson, Tom and Brendon Conlan; December, 2008, at Tidbinbilla Nature Reserve in Canberra for the group Christmas party. **b** Student dinner at Tom’s house viewing some photographic slides from Tom’s travels. From left to right: Adele Williamson, Carly Smith, Brendon Conlan, Iain McConnell, Rhys Jones and Tom

Tom looked after his students and post-doc’s well-being and was keen to associate with them outside of the working hours. Tom loved scientific discussions, and facilitated them by always being available, being an excellent listener, and by finding new interesting angles for looking at results or problems. Also, his knowledge about the history of photosynthesis research, and the people involved, was highly fascinating. It was these excellent discussions with Tom and his infectious love for experiments that made Johannes and his young family move from Berlin to Canberra, Australia, so that Johannes could join Tom. When Johannes arrived with his family in Canberra, Tom not only picked them up from the airport, but also hosted them in his house until they found an apartment. Johannes and his family also fondly remember several trips to the Australian coastlines, to the nature reserve Tidbinbilla near Canberra, and the 2800 km journey in Tom’s car from Canberra to Uluru (Ayers Rock) in the Red Center of Australia. For all his co-workers, Tom often put on video or beer and pizza nights at his place (Fig. 6a, b). Tom was renowned for putting on the best snacks menu around, offering so much food that rarely anyone could try all the pizzas ordered.

Tom will be fondly remembered and sadly missed by those who knew him. The widespread appreciation of Tom is evident in the recollections of colleagues collected and published (see Govindjee 2008; Govindjee et al. 2018).
